# Oral Semaglutide under Human Protocols and Doses Regulates Food Intake, Body Weight, and Glycemia in Diet-Induced Obese Mice

**DOI:** 10.3390/nu15173765

**Published:** 2023-08-28

**Authors:** Yermek Rakhat, Lei Wang, Wanxin Han, Aktolkyn Rustemova, Nazymgul Kulzhanova, Yuichiro Yamada, Daisuke Yabe, Yutaka Seino, Toshihiko Yada

**Affiliations:** 1 Division of Integrative Physiology, Kansai Electric Power Medical Research Institute, Kyoto 604-8436, Japan; rakhatyermek@gmail.com (Y.R.);; 2 Department of Diabetes, Endocrinology and Metabolism/Rheumatology and Clinical Immunology, Gifu University Graduate School of Medicine, Gifu 501-1194, Japan; 3 Yutaka Seino Distinguished Center for Diabetes Research, Kansai Electric Power Medical Research Institute, Osaka 553-0003, Japan; 4 Center for One Medicine Innovative Translational Research, Gifu University Institute for Advanced Study, Gifu 501-1193, Japan

**Keywords:** oral semaglutide, GLP-1 receptor agonist, obesity, diabetes, food intake, body weight, blood glucose, DIO mice

## Abstract

The first oral form of the glucagon-like peptide-1 receptor agonist, oral semaglutide, has recently been launched and potently controls glycemia and body weight in subjects with type 2 diabetes. This drug carries the absorption enhancer and requires specific protocols of administration. The mechanism of action of oral semaglutide is not fully understood, for which an appropriate experimental model is required. This study explores the metabolic effects of oral semaglutide in mice under human protocols and doses. Oral semaglutide was bolus and once daily injected into high-fat diet-induced obese (DIO) mice under human protocols, followed by monitoring blood glucose, food intake, and body weight. Oral semaglutide 0.23 mg/kg, a comparable human dose (14 mg) in a small volume of water under human protocols rapidly decreased blood glucose and food intake and continuously reduced food intake and weight gain for 3 days in DIO mice. At 0.7 mg/kg (42 mg), this drug was somewhat more potent. Oral semaglutide with human protocols and doses rapidly reduces blood glucose and food intake and continuously suppresses feeding and weight in DIO mice. This study establishes mice as a model suitable for analyzing the mechanism of anti-obesity/diabetes actions of oral semaglutide.

## 1. Introduction

Obesity and diabetes are serious worldwide health problems. However, safe and orally effective drugs to treat obesity have long been unavailable. Recently, the first oral form of glucagon-like peptide-1 receptor agonist (GLP-1RA) semaglutide (Rybelsus^®^) has been launched as a treatment for type 2 diabetes [1] and shown to potently ameliorate obesity as well as diabetes [2]. Oral semaglutide carries the absorption enhancer sodium *N*-[8-(2 ydroxybenzoyl)amino] caprylate (SNAC), providing the absorptive capacity of this medicine [3]. Oral semaglutide 3, 7, and 14 mg doses are approved for clinical use [1]. In the PIONEER 1 study with 703 type 2 diabetic subjects, 7 and 14 mg of oral semaglutide significantly reduced hemoglobin A1C (HbA_1C_) at 26 weeks and body weight at 52 weeks of treatment [4]. Treatment with oral semaglutide 14 mg for 12 weeks reduced energy intake and body fat mass in association with increased satiety and fullness, in subjects given fat-rich (high-calorie) breakfasts [2]. Oral semaglutide reduces body weight to a greater extent than dulaglutide and liraglutide, other GLP-1RAs [5,6]. PIONEER trials have shown outstanding efficacy of oral semaglutide in glycemic and weight control [7]. 

Successful absorption of oral semaglutide in the stomach requires special cautions: an overnight-fasted and drinking-restricted state and early morning administration of the drug with a small volume of water (<120 mL), followed by an additional fasted and drinking-restricted state for 30 min [8]. These protocols are thought to support the absorption and action of this drug. However, the mechanisms of action of oral semaglutide remain to be fully elucidated, for which an appropriate experimental model is needed. It has been documented that the anatomical, physiological, and biochemical differences in the gastrointestinal tract of humans and common laboratory animals can cause significant variations in drug absorption from the oral route [9]. For instance, the transit time can be significantly different between species due to different dimensions and propulsive activities of the gastrointestinal tract [9]. Therefore, the selection of the right animal model is important [9]. The mouse is a well-standardized and stable laboratory animal used widely in the world. However, the effectiveness of oral semaglutide in mice remains unknown. In addition, in some anti-diabetic drugs, a 10–1000-fold higher concentration is required in mice than in humans [10,11,12,13], indicative of lower drug sensitivity in mice than humans. 

The present study aims to explore the effects of oral semaglutide with human protocols and doses on food intake, body weight, and blood glucose in DIO C57BL/6J mice. The results show that the metabolic effects of oral semaglutide reported in humans are reproduced in DIO mice provided the human protocols, including doses and restricted water volume, are used. 

## 2. Materials and Methods

### 2.1. Chemicals

Oral semaglutide (Rybelsus^®^) was purchased from Novo Nordisk (Copenhagen, Denmark).

### 2.2. Animals 

Male C57BL/6J mice aged 4 weeks were obtained from Japan SLC (Shizuoka, Japan) and fed with high-fat diet (D12492) (Research Diets, Inc., New Brunswick, NJ, USA) for 30–36 weeks. They were housed under controlled temperature (23 ± 1 °C) and humidity (55 ± 5%) with 12 h light/dark cycle (light on at 8:00 and off at 20:00). High-fat diet fed DIO mice with an average body weight of around 50–55 g were used in the present experiments. DIO mice that showed abnormal feeding behavior (overeating/less eating/wasting food) were excluded from experiments at the very initial point (handling). Mice were housed in grouped cages before handling, then moved to single cages from the handling period through the end of experiments. Animal experiments were carried out after receiving approval from the Institutional Animal Experiment Committee and in accordance with the Institutional Regulation for Animal Experiments at Gifu University.

### 2.3. Study Design

**Figure 1:**
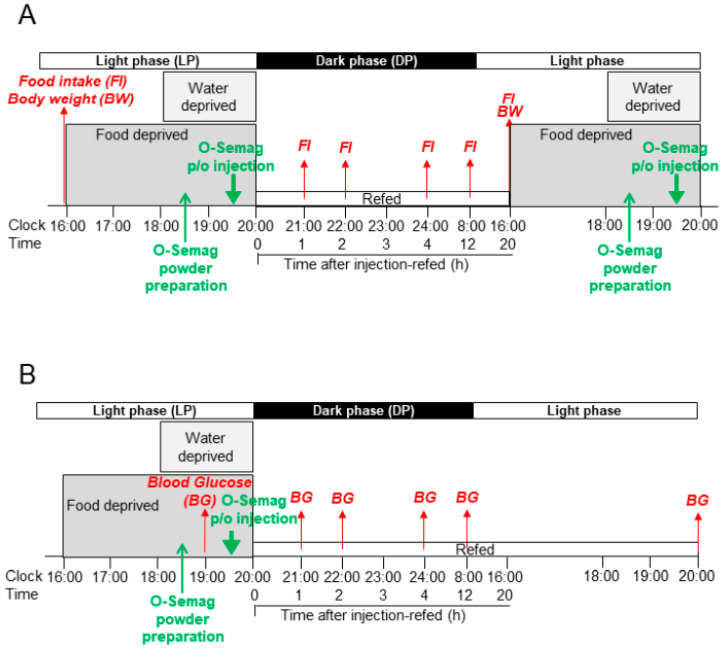
(**A**) After measuring food intake (*FI*) and body weight (*BW*) at 16:00, food was deprived from 16:00 for 3.5 h and water was deprived from 18:00 for 1.5 h prior to injection of oral semaglutide at 19:30. An oral semaglutide pill was crushed into powder at 18:30. Oral semaglutide or control DW was p/o injected at 19:30, the circadian time of the start of activities in mice, which corresponds to the early morning (7:30) in humans. Subsequently, food and water were deprived for additional 0.5 h until 20:00, and refeeding started at 20:00 (injection-refed). *FI* was measured at 21:00, 22:00, 24:00, and at 08:00 and 16:00 the next day. In the sub-chronic experiments with once-daily oral semaglutide injection for 3 days, food intake was measured at 8:00 and 16:00 to assess dark phase (DP) and light phase (LP) food intake. *BW* was measured once daily at 16:00 from day 1 to day 3. (**B**) Measurement of blood glucose (*BG*) was performed with the procedure described in panel (**A**) except that *BG* prior to oral semaglutide injection was monitored at 19:00, followed by measurements at 21:00, 22:00, 24:00, and 08:00 and 20:00 the next day.

**Figure 2:**
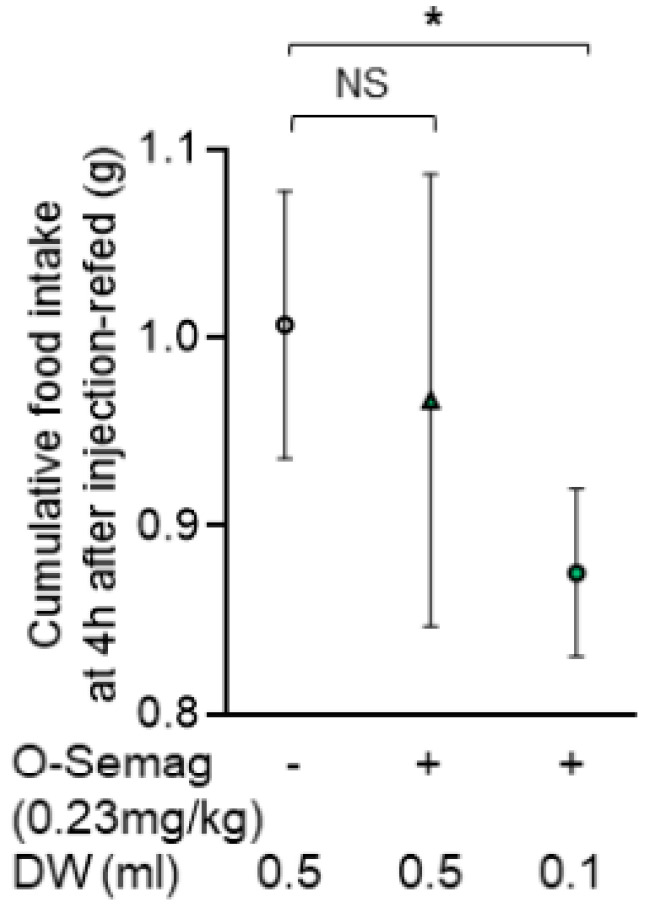
**Water volume-dependent effect of oral semaglutide on food intake in DIO mice.** Under the protocol described in Figure 1A, oral semaglutide 0.23 mg/kg (eq. 14 mg) with 0.5 mL DW, compared with 0.5 mL DW, did not significantly alter cumulative food intake at 4 h after injection-refed. Oral semaglutide 0.23 mg/kg (eq. 14 mg) with 0.1 mL DW significantly reduced cumulative food intake at 4 h after injection-refed. All data are presented as mean ± SEM. * *p <* 0.05 by one-way ANOVA followed by Dunnett’s multiple comparisons test. n = 7 mice in each group. NS; not significant.

**Figure 3:**
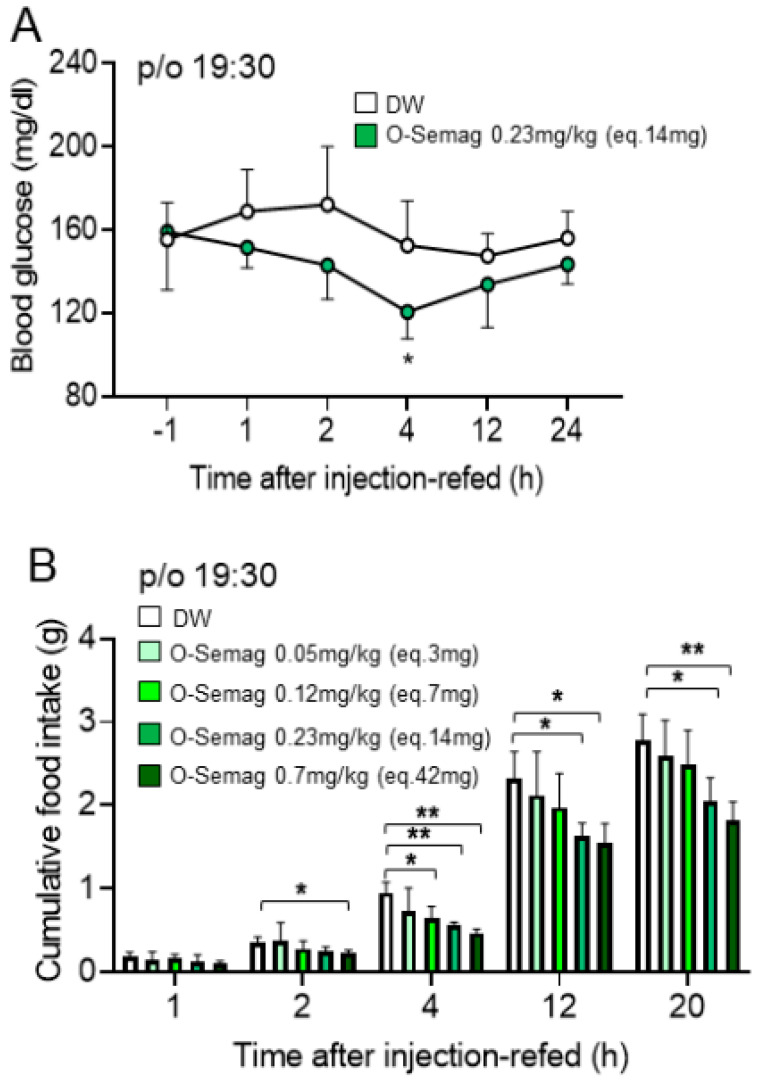
**Effects of oral semaglutide on blood glucose and cumulative food intake in DIO mice.** (**A**) Oral semaglutide 0.23 mg/kg (eq. 14 mg) with 0.1 mL DW significantly decreased blood glucose at 4 h but not at 1, 2, 12, or 24 h after injection-refed. (**B**) Oral semaglutide 0.23 mg/kg (eq. 14 mg) with 0.1 mL DW and 0.7 mg/kg (eq. 42 mg) with 0.1 mL DW significantly reduced cumulative food intake at 4, 12, and 20 h after injection-refed, and at 0.7 mg/kg (eq. 42 mg) with 0.1 mL DW, it additionally reduced cumulative food intake at 2 h. At a lower dose of 0.12 mg/kg (eq. 7 mg) with 0.1 mL DW, oral semaglutide reduced cumulative food intake only at 4 h after injection-refed, and at 0.05 mg/kg (eq. 3 mg) with 0.1 mL DW it did not alter cumulative food intake at any time point. All data are presented as mean ± SEM. * *p <* 0.05 and ** *p * < 0.01 by two-way RM ANOVA followed by Dunnett’s multiple comparisons test. n = 7 in each group (**A**), n = 6 in each group (**B**).

**Figure 4:**
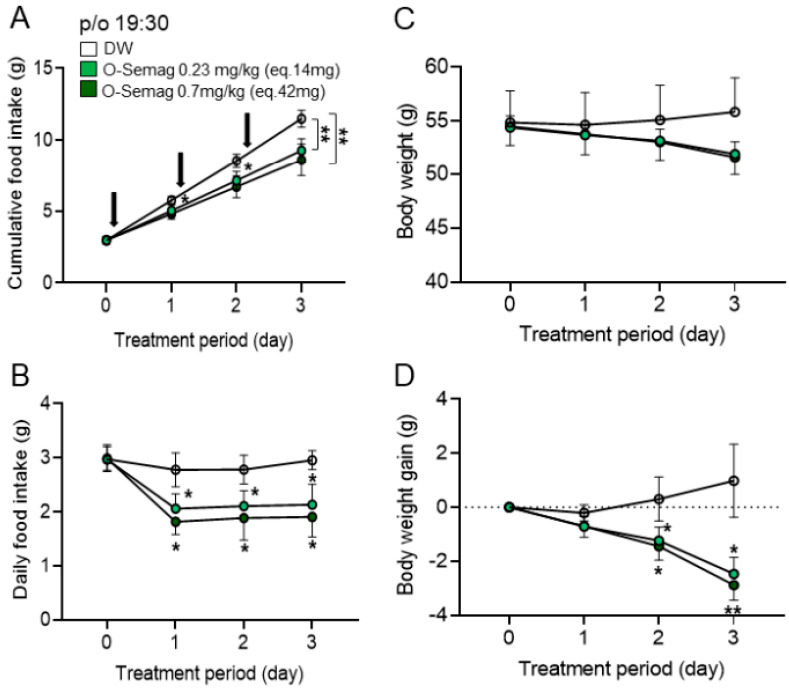
**Sub-chronic effect of oral semaglutide on food intake and body weight in DIO mice.** (**A**,**B**) Oral semaglutide at 0.23 mg/kg (eq. 14 mg) with 0.1 mL DW and 0.7 mg/kg (eq. 42 mg) with 0.1 mL DW significantly reduced cumulative food intake (**A**) and daily food intake (**B**) at days 1, 2, and 3 of the treatment. (**C**,**D**) Oral semaglutide at 0.23 mg/kg (eq. 14 mg) with 0.1 mL DW and 0.7 mg/kg (eq. 42 mg) with 0.1 mL DW had no significant effect on body weight (**C**) but significantly decreased body weight gain (**D**) in DIO mice on days 2 and 3 of the treatment. Dotted line; the level just before treatment on day 0. All data are presented as mean ± SEM. * *p <* 0.05 and ** *p <* 0.01 compared to DW, by two-way RM ANOVA followed by Sidak’s multiple comparisons test. n = 6 in each group.

**Figure 5:**
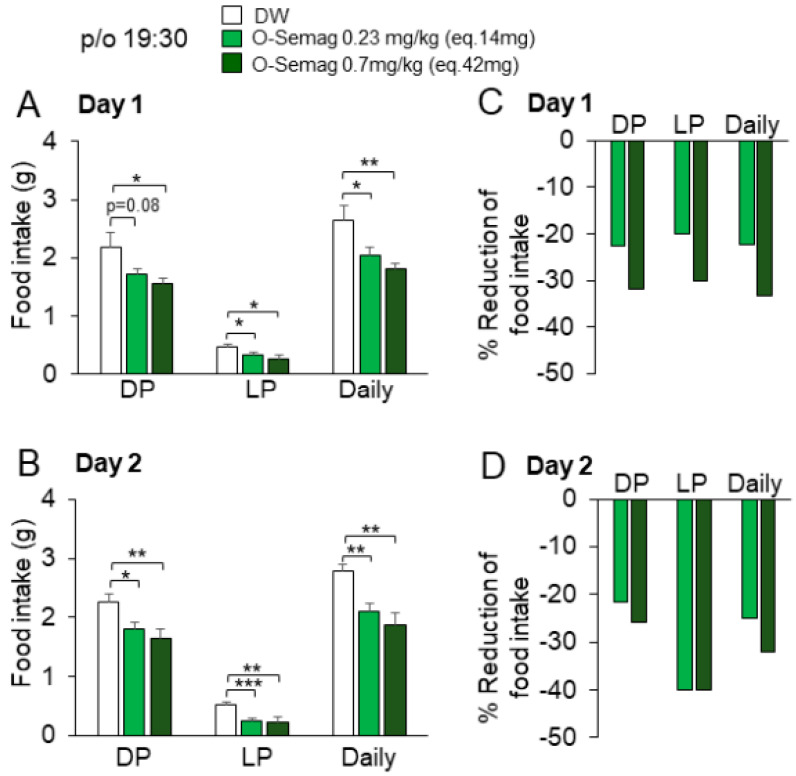
**Effect of oral semaglutide on circadian feeding behavior in DIO mice.** (**A**) On day 1 of the treatment, oral semaglutide 0.23 mg/kg (eq. 14 mg) with 0.1 mL DW significantly reduced LP and daily food intake, and at 0.7 mg/kg (eq. 42 mg) with 0.1 mL DW, it also significantly reduced DP food intake. (**B**) On day 2 of the treatment, DP, LP, and daily food intake were significantly reduced by oral semaglutide at 0.23 mg/kg (eq. 14 mg) with 0.1 mL DW and 0.7 mg/kg (eq. 42 mg) with 0.1 mL DW. (**C**,**D**) Percent reductions of DP, LP, and daily food intake by oral semaglutide on day 1 (**C**) and day 2 (**D**). Percent reductions of LP food intake by oral semaglutide at 0.23 mg/kg (eq. 14 mg) with 0.1 mL DW and 0.7 mg/kg (eq. 42 mg) with 0.1 mL DW on day 2 were larger than those on day 1. All data are presented as mean ± SEM. * *p <* 0.05, ** *p <* 0.01, and *** *p <* 0.001 by two-way RM ANOVA followed by Tukey’s multiple comparisons test. n = 6 in each group.

Experimental design is shown in Figure 1. In the experiments presented in Figure 2, male DIO mice (age 30 weeks, average body weight (BW) 50g) were divided according to their food intake into three groups (n = 7), which were p/o given oral semaglutide 0.23 mg/kg with 0.5 or 0.1 mL DW in test groups and with 0.5 mL DW in control groups. In experiments presented in Figure 3A, DIO mice (age 32 weeks, average BW 50 g) were divided into two groups (n = 7) according to blood glucose, then oral semaglutide 0.23 mg/kg with 0.1 mL DW for test groups and 0.5 mL DW for control groups were p/o injected. In experiments presented in Figure 3B, DIO mice (age 32 weeks, average BW 50 g) were divided into 5 groups (n = 6) according to daily food intake, and oral semaglutide at four different doses with 0.1 mL DW for test groups and 0.5 mL DW for control groups were p/o injected. In experiments presented in Figure 4 and Figure 5, DIO mice (age 36 weeks, average BW 55 g) were divided into 3 groups (n = 6) according to both body weight and food intake, then oral semaglutide 0.23 and 0.7 mg/kg with 0.1 mL DW for test groups and 0.5 mL DW for control groups were p/o injected. 

### 2.4. Oral Semaglutide Dose Selection, Preparation and Protocol of p/o Injection

Oral semaglutide 3, 7, and 14 mg are approved for humans [1]. Oral semaglutide 7 and 14 mg provide adequate systemic exposure over the body weight range of 40–188 kg [14]. Our study aimed to use the oral semaglutide doses equivalent to (eq.) these clinical doses in humans. Oral semaglutide 3, 7, and 14 mg divided by 60 kg, a common body weight, were used: 0.05 mg/kg (eq. 3 mg), 0.12 mg/kg (eq. 7 mg), and 0.23 mg/kg (eq. 14 mg). A threefold higher dose, 0.7 mg/kg (eq. 42 mg), was also examined. On experimental days at 18:30, a fresh pill of oral semaglutide was crushed in the special porcelain “pill crusher” by rotating the crusher 20–25 times each to the right and left to yield powder (Figure 1A,B). A solution containing oral semaglutide powder in distilled water (DW) was shaken 8–10 times by hand and p/o injected into DIO mice using a stainless needle syringe. Mice were deprived of food at 16:00 and of water at 18:00, p/o injected with oral semaglutide or DW at 19:30, and food and water were returned at 20:00 (defined as “injection-refed” time) (Figure 1A,B). Mice had free access to water and food from 20:00∼16:00 next day.

### 2.5. Measurement of Blood Glucose, Food Intake, and Body Weight

Blood was sampled from mice fasted 3 h at 19:00, 0.5 h before injection of oral semaglutide, and blood glucose levels were measured with GLUCOCARD PlusCare GT-1840 (ARKRAY Factory, Inc., Koka, Shiga, Japan) as reported [12]. Subsequently, cumulative food intake (FI) was measured at 1, 2, 4, 12, and 20 h after injection-refed (Figure 1A) and blood glucose (BG) was measured at 1, 2, 4, 12, and 24 h after injection-refed (Figure 1B). In sub-chronic experiments with once-daily injection for 3 days, daily food intake and body weight (BW) were measured at 16:00 (Figure 1A). Food intake in the light phase (LP) from 8:00 to 16:00 before fasting and that in the dark phase (DP) from 20:00 to 8:00 the next day were also measured. 

### 2.6. Statistical Analysis 

All data are expressed as means ± SEM. Statistical analysis was performed by unpaired *t*-test, two-way RM ANOVA followed by Dunnett’s, Sidak’s, or Tukey’s multiple comparisons test. All statistical analyses were performed using Prism 9 (GraphPad Software, San Diego, CA, USA). *p* < 0.05 was considered significant. 

## 3. Results 

### 3.1. Effect of Oral Semaglutide with Different Water Volumes on Food Intake

Under the protocol described in Figure 1A, DW 0.5 mL (control), oral semaglutide 0.23 mg/kg (eq. 14 mg) with 0.5 mL DW and that with 0.1 mL DW were p/o administered at 19:30 in DIO mice fasted 3.5 h and water-deprived for 1.5 h, followed by 30 min post-treatment deprivation of food and water. Oral semaglutide 0.23 mg/kg (eq. 14 mg) in 0.5 mL DW, compared to the control with 0.5 mL DW, did not significantly alter cumulative food intake, including that at 4 h after injection-refed in DIO mice (Figure 2). In contrast, oral semaglutide 0.23 mg/kg (eq. 14 mg) in 0.1 mL DW significantly decreased cumulative food intake at 4 h after injection-refed. These data show that preparing oral semaglutide in a small water volume of 0.1 mL is essential for this medicine to exert effects in mice.

### 3.2. Acute Effect of Oral Semaglutide 0.23 mg/kg (Eq. 14 mg) on Blood Glucose in DIO Mice

Under the protocol described in Figure 1B, oral semaglutide 0.23 mg/kg (eq. 14 mg) and DW were p/o administered at 19:30 in DIO mice fasted 3.5 h and water-deprived for 1.5 h, followed by 30 min post-treatment deprivation of food and water, and blood glucose (BG) was measured at 1, 2, 4, 12, and 24 h after injection-refed. Oral semaglutide significantly decreased blood glucose at 4 h after injection-refed, while blood glucose at 1, 2, 12, and 24 h was not significantly altered (Figure 3A). The result shows that oral semaglutide under human clinical protocol and dose acutely lowers blood glucose in DIO mice. 

### 3.3. Dose-Dependent Effect of Oral Semaglutide on Cumulative Food Intake in DIO Mice

Under the protocol described in Figure 1A, oral semaglutide 0.23 mg/kg (eq. 14 mg) significantly inhibited cumulative food intake at 4, 12, and 20 h, but not at 1 and 2 h after injection-refed (Figure 3B). At 0.7 mg/kg (eq. 42 mg), a dose threefold higher than a human dose, cumulative food intake was reduced at 4, 12, and 20 h to a somewhat larger extent and reduced additionally at 2 h (Figure 3B). At a lower dose of 0.12 mg/kg (eq. 7 mg), oral semaglutide significantly decreased cumulative food intake only at 4 h, while at 0.05 mg/kg (eq. 3 mg), it did not alter cumulative food intake at any time point (Figure 3B). Thus, oral semaglutide at doses of 0.23 mg/kg (eq. 14 mg) and 0.7 mg/kg (eq. 42 mg) markedly and long-lastingly suppressed food intake in DIO mice.

### 3.4. Sub-Chronic Effect of Oral Semaglutide on Food Intake and Body Weight in DIO Mice

Following the protocol described in Figure 1A, the sub-chronic effect of oral semaglutide was examined by once-daily p/o injection at 19:30 for 3 days in DIO mice. Oral semaglutide at 0.23 mg/kg (eq. 14 mg) and 0.7 mg/kg (eq. 42 mg) compared to DW significantly decreased cumulative (Figure 4A) and daily food intake (Figure 4B) at day 1 to day 3 of the treatment. The body weight of DIO mice was not significantly altered by oral semaglutide at the two doses (Figure 4C). Body weight gain was significantly reduced by oral semaglutide (Figure 4D): the reductions with 0.23 mg/kg (eq. 14 mg) and 0.7 mg/kg (eq. 42 mg) were, respectively, 1.2 g and 1.4 g on day 2 and 2.4 g and 2.7 g on day 3 of the treatment, and the corresponding percent reductions were, respectively, 2.18% and 2.54% on day 2 and 4.36% and 4.90% on day 3 of the treatment. Thus, the extent of weight reduction was greater on day 3 than on day 2, and on day 3 it was larger with the higher dose. These results show that oral semaglutide constantly decreases daily food intake and progressively reduces body weight gain during the treatment period of 3 days in DIO mice.

### 3.5. Effect of Oral Semaglutide on Diurnal Food Intake in DIO Mice

Under the protocol described in Figure 1A, oral semaglutide 0.23 mg/kg (eq. 14 mg) was injected at 19:30 to DIO mice with average body weight around 55 g, approximately 10% heavier than that of DIO mice used in other experiments. It significantly reduced food intake in LP (8:00 next day~16:00 next day) but not DP (20:00~8:00 next day) (*p* = 0.08) on day 1 of the treatment (Figure 5A), whereas it significantly reduced DP food intake in DIO with body weight around 50 g (Figure 3B). These results indicate that the severity of obesity may influence the ability of this medicine to reduce DP food intake. On day 2, oral semaglutide reduced LP food intake to a greater extent than DP food intake (Figure 5B), showing a preferential suppression of LP food intake over DP food intake in DIO mice with relatively severer obesity. At a higher dose of 0.7 mg/kg (eq. 42 mg), oral semaglutide reduced both LP and DP food intake on day 1 and day 2 (Figure 5A,B). At the two doses, percent reductions of LP food intake on day 2 were larger than those on day 1 (Figure 5C,D), suggesting that the effect progressed during the initial 2 days of treatment. 

## 4. Discussion

The present study demonstrated that oral semaglutide injected under human protocols and doses is effective in DIO mice. Oral semaglutide injected with 0.1 mL water lowered glycemia, food intake, and body weight, while a five times larger volume (0.5 mL) of water blunted the effect of this drug on food intake. An amount of 0.1 mL water per mouse with a body weight of 50 g (0.2% body weight) corresponds to the human protocol of 60–120 mL water per subject with a body weight of 60 kg (0.1–0.2% body weight). These data show that the caution in clinical practice to take oral semaglutide with a small volume of water for successful absorption and effectiveness of this medicine applies to mice. Whether the small water volume and/or high concentration of this medicine in the stomach is essential for its absorption and effectiveness remains unclear and requires future study. Oral semaglutide 0.23 mg/kg, a comparable human dose (14 mg) injected into DIO mice after fasting and water deprivation for several hours, following human protocols, rapidly decreased blood glucose and cumulative food intake, reaching significant levels at 4 h after injection-refed. 

Moreover, oral semaglutide at 0.23 mg/kg injected once daily for 3 days reduced LP and daily food intake on days 1–3 and body weight gain on days 2~3 of the treatment, and at a higher dose of 0.7 mg/kg (eq. 42 mg) it elicited these effects to somewhat greater extents and additionally reduced DP food intake, consistent with the report in humans [15]. This dose dependency is of relevance since the use of semaglutide at a higher dose of 50 mg is under consideration for treating obese subjects [16]. Thus, the present study demonstrates that the metabolic effects of oral semaglutide reported in humans [3,4,5,6,7,17,18] are reproduced in mice provided the human protocols and doses are used. This finding establishes mice as a suitable model for exploring the mechanism of action of oral semaglutide, which enables the mouse studies aimed at providing a solid scientific basis for the treatment of obesity and diabetes with this new medicine.

In PIONEER 10 studies, the 14 mg dose of oral semaglutide was effective in reducing HbA_1c_ and body weight [6]. In the present study, oral semaglutide 0.23 mg/kg (eq. 14 mg) acutely and significantly reduced blood glucose and food intake at 4 h after injection-refed and, when once-daily administered, continuously reduced food intake on days 1~3 and body weight gain on days 2~3 in DIO mice. Oral semaglutide at 0.7 mg/kg (eq. 42 mg), a dose threefold higher than the highest human dose (eq. 14 mg), reduced food intake and body weight to a slightly larger extent. Thus, the most effective dose range of oral semaglutide for reducing body weight in DIO mice appears to be 0.23 mg/kg (eq. 14 mg) ~0.7 mg/kg (eq. 42 mg), a range severalfold higher than that used to treat diabetes in humans. This result is consistent with the human study [15] showing that levels of circulating semaglutide determine reductions in HbA1c and body weight and the dose required for reducing weight is severalfold higher than that for lowering HbA_1C_. In this regard, the use of a severalfold higher concentration of semaglutide for treating obese subjects is under consideration in the US [16]. Our data that oral semaglutide at a threefold higher concentration is more efficacious in lowering feeding and weight provides scientific validity for this consideration. The mechanism underlying the difference in effective doses for lowering body weight vs. blood glucose remains to be elucidated.

In the present study, the reduction of body weight in oral semaglutide-treated DIO mice was −4.90% (−2.7 g/55 g BW) on day 3 of the treatment. This relative reduction is comparable to −3.03% (−2.2 kg/72.7 kg BW) [5] and −4.68% (−4.4 kg/94.0 kg BW) [4] at 26 weeks of the treatment with oral semaglutide in human trials. Clinical studies in obese and overweight Japanese people (n = 3480) using a nationwide intervention program database have shown that a 3% weight reduction in 6 months is effective in ameliorating obesity-related risk factors [14,19]. Therefore, the 4.90% body weight reduction by oral semaglutide observed in the present study may be related to the amelioration of obesity-related conditions. 

LP hyperphagia is causally related to obesity in mice and rats [11,20], and restriction of feeding during LP ameliorates obesity and related diseases [11,12]. In the present study, once-daily administration of oral semaglutide at a human dose (0.23 mg/kg; eq. 14 mg) preferentially reduced LP food intake in DIO mice with advanced weight gain by 10%. Furthermore, at this (0.23 mg/kg) and higher (0.7 mg/kg) doses, percent reductions of LP food intake were larger on day 2 than on day 1, suggesting that the effect of oral semaglutide to suppress LP food intake progresses during initial 2 days of treatment. The ability of oral semaglutide to preferentially and stably suppress LP food intake, a newly found property of this drug, could serve to effectively lower body weight in obesity.

Anti-diabetic drugs reportedly lower blood glucose in mice at 10–1000-fold higher concentrations than those in humans [13,21,22,23] or are even ineffective in mice [24], indicating lower drug sensitivity in mice than humans. This different drug sensitivity may be related to the different feeding behaviors: humans usually eat three times a day, whereas mice eat continuously when they are awake. Hence, subjects treated with anti-diabetic drugs are usually instructed to keep the right feeding pattern. However, humans with irregular lifestyles and/or prolonged work, such as shift workers and taxi drivers, experience irregular feeding behavior, which possibly reduces the drug efficacy. The present study demonstrates that oral semaglutide at human clinical doses is effective in mice under free access to food, provided they are kept from eating and drinking for only a few hours when the drug is administered. Whether this distinct property of oral semaglutide is related to its absorption by the stomach as well as the nature of the semaglutide molecule remains to be clarified. Our results suggest that oral semaglutide can act irrespective of the feeding pattern and hence be used to treat a wider range of subjects, including those with irregular lifestyles and feeding behaviors.

## Data Availability

Not applicable.
